# Analysis of the Economic Impact of Cardiovascular Diseases in the Last Five Years in Brazil

**DOI:** 10.5935/abc.20170068

**Published:** 2017-07

**Authors:** Alessandra de Sá Earp Siqueira, Aristarco Gonçalves de Siqueira-Filho, Marcelo Gerardin Poirot Land

**Affiliations:** Universidade Federal do Rio de Janeiro, Rio de Janeiro, RJ - Brazil

**Keywords:** Cardiovascular Diseases / economy, Cadiovascular Diseases / mortality, Costs and Cost Analysis, Hospitalization

## Abstract

**Background:**

There is growing concern about the economic impact of cardiovascular diseases (CVD) in Brazil and worldwide.

**Objective:**

To estimate the economic impact of CVD in Brazil in the last five years.

**Methods:**

The information to estimate CVD costs was taken from national databases, adding the direct costs with hospitalizations, outpatient visits and benefits granted by social security. Indirect costs were added to the calculation, such as loss of income caused by CVD morbidity or mortality.

**Results:**

CVD mortality accounts for 28% of all deaths in Brazil in the last five years and for 38% of deaths in the productive age range (18 to 65 years). The estimated costs of CVD were R$ 37.1 billion in 2015, a 17% increase in the period from 2010 to 2015. The estimated costs of premature death due to CVD represent 61% of the total cost of CVD, Direct costs with hospitalizations and consultations were 22%, and costs related to the loss of productivity related to the disease were 15% of the total. Health expenditures in Brazil are estimated at 9.5% of GDP and the average cost of CVD was estimated at 0.7% of GDP.

**Conclusion:**

CVD costs have increased significantly in the last five years. It is estimated that CVD costs increase as the Brazilian population ages and the prevalence of CVD increases.

## Introduction

Non-communicable chronic diseases (CDNs) - mainly cardiovascular disease (CVD), cancer, chronic respiratory diseases - are the leading cause of death, causing approximately 38 million deaths annually worldwide.^1^ Approximately 82% of premature deaths from non-communicable diseases occur in low- and middle-income countries, which can be largely avoided. Statistics show that approximately half of these deaths occur during the productive life of individuals^2^ and CVD accounts for most of them, accounting for 37%.^1^

The socioeconomic impact of chronic diseases is increasing and is considered a problem for the world public health. In addition to premature deaths, NCDs are responsible for incapacity for work, reduction of family incomes and reduction of productivity.^3,4^

Current health spending in Brazil is approximately 9.5% of GDP per capita. Data from the World Health Organization indicate health expenditures of $ 1078 per capita in 2012 in Brazil. Of these 47.5% are financed by the government, which corresponds to 7.9% of the total expenditure of the Brazilian government.^5^ In this same period, developed countries spend an average of 4632 dollars per capita, that correspond to the average of 16,8% of the governmental expenses with health.

The cost of hospitalizations for cardiovascular diseases is considered the largest cause of hospital admissions in Brazil^6^ and recent IBGE data show that Brazil is changing its age structure very rapidly, increasing the proportion of elderly people and life expectancy of the Brazilian.^7^ Aging tends to increase the incidence of CVD and, consequently, its costs exponentially.^8^

This study was designed to estimate the economic impact of CVD based on Brazilian data. Our estimate of socioeconomic impact is based on government expenditures in Brazil, once the information from the public database of the health system was used. The methodology proposed in the present study includes the direct and indirect costs related to CVD.

## Methods

Health costs can be divided into:

1. Direct costs: costs of direct **medical care** to the patient, such as medical services rendered and treatments performed, and **non-medical costs** (non-medical visits);^9^
2. Indirect costs: costs of morbidity and mortality. **Morbidity costs** are defined as expenses for the temporary or permanent loss of work activities due to the disease studied. **Mortality costs** are the costs estimated for premature death as a consequence of illness.^1;9^

### Data Sources

Data sources are publicly available information on the Hospital Mortality System (SIM),^10^ the hospital morbidity was obtained at the approved hospital admission events of the DATASUS Hospital Information System (SIH) and outpatient information system (SIA),^11^ in addition to information on social security expenses for temporary and / or permanent removal - DATAPREV.^12^

In order to estimate the total cost of diseases in Brazil, information from previous observational studies^13^ was collected, as well as from the access to the World Health Organization (WHO) database.^14^ To estimate the costs of private care, the sources of information in the National Health Agency (ANS) were used. In order to evaluate the impact of the cost of mortality, estimates from the Brazilian Institute of Geography and Statistics (IBGE) were used, such as: population estimates, life expectancy by sex and age group, average salary of the Brazilian population, and rate of unemployment.^7^ A productive range was considered from 18 to 65 years.

### Hospital Admissions

The number of hospital admissions for CVD is available in the billing data approved in the SIH-SUS,^11^ and corresponds to all events registered in Chapter IDC-10: IX Circulatory diseases in the SUS.^15^ To estimate the number of hospital admissions in private care, public data banks (ANS)^16^ were used, which show the coverage rate of the beneficiaries of the private health plans, that is, the percentage of beneficiaries who use private plans each year. The formula used to estimate the number of admissions to supplementary care was:

Number of hospitalizations of the supplementary care

$$=\frac{\mathrm{number}\;\mathrm{of}\;\mathrm{hospitalizations}\;\mathrm{SUS}\times \mathrm{coverage}\;\mathrm{rate}\;\mathrm{ANS}}{100-\mathrm{ANS}\;\mathrm{coverage}\;\mathrm{rate}}$$

The number of medical consultations performed by CVD was estimated through the Outpatient Production of the SUS - Brazil - by location of care, available in the SIA / SUS.^11^ We extracted the information of quantities approved in the Subgroup procedure: 0301 Consultations / Attendance / Monitoring. As the number of consultations performed by ICD of illness is not available in the health information system, in the present study the percentage of hospitalizations for CVD was calculated on the total hospitalizations. This percentage value was applied in the total number of consultations performed in the SUS (10% of the total), since it may be underestimated. To estimate the number of outpatient visits performed in the private sector, the coverage rate of the ANS was applied.

SUS referrals to private sector beneficiaries are accounted by the ANS^16^ and reimbursement amounts for hospitalizations and/or outpatient visits were accounted for the direct costs calculations.

### Direct costs

The direct costs were calculated in Brazilian currency (reais) for the year 2016.

The hospital costs related to CVD events were separated in didactic form in:

1. Clinical treatments of CVD - subgroup 0303, titled "Clinical treatments (other specialties)";
2. Surgical treatments of CVD - subgroup 0406, entitled "Circulatory System Surgery" - which include: cardiological surgical procedures, arrhythmia procedures (pacemakers, cardiac defibrillators and electrophysiological studies - FES), coronary angioplasty, and vascular (surgical and / or percutaneous).^11^

The costs of surgical hospitalizations were increased by estimates of costs of orthotics, prostheses or special materials (OPME). In order to estimate costs incurred with OPME, is used the price base available at ANVISA, with the lowest price practiced in Brazil for each type of OPME.

### Indirect costs

Indirect costs are calculated through the costs of morbidity (loss of productivity caused by CVD) together with the costs of mortality, cost of CVD premature death.^17^ The costs of morbidity can be defined as time and economic production lost by the patient's absence from their usual activities and work as a direct result of CVD or their treatment.^18^ The calculations were grouped into two components:

1. Costs for temporary removals from the work of the patients employed (absenteeism): in this component are included the removals for hospitalizations and medical consultations, and added the values spent in the displacement, that is, the transportation for each consultation carried out;
2. Costs of patients who are no longer in working condition as a consequence of CVD (permanent or temporary removals paid by the government). Costs of pensions and sickness-help caused by CVD.

### Social security benefits

The information available on the Social Security website was used to estimate the impact of temporary (sickness) and permanent (retired) removal. An analysis was made of the number of benefits granted because of CVD excluding benefits from other diseases outside the ICD-10 Chapter IX. Diseases of the circulatory system.^12^

**Figure 1:**
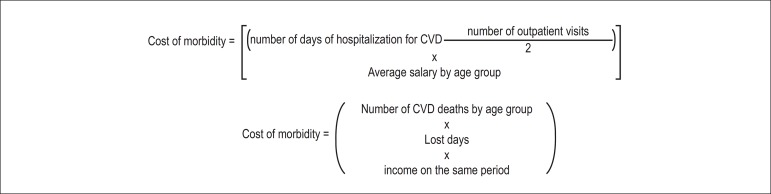
Formulas used to estimate cost.

The benefits granted by Social Security were analyzed by the frequency of events in the sample in the CVD disease group in the period from 2008 to 2013. For the estimation of removals occurred in the years 2014 and 2015, a projection was made based on the time series of the period from 2008 to 2013 from a model (ARIMA). The ARIMA model is a type of moving average model. A methodology widely used for the elaboration of forecasting studies, applied in the spss software forecasting module.^19^ The benefits granted because of CVD correspond to 25% of the total expenses granted by the Social Security.Mortality costs are estimated by estimating the years of productive life lost due to premature death as a consequence of CVD. The calculation is done by multiplying the number of deaths because of CVD according to the age group by the number of days lost (difference between the life expectancy of the Brazilian population and the age of premature death).^17^ This data is expressed in economic value multiplying the days lost by the estimated income of the Brazilian until the age of 65 years. Figure 1. In the present study, the salary used for the calculation was the average salary of the Brazilian population, corrected by the rate of unemployment in the same period.^7^

## Results

Table 1 shows that there was an increase of 3% of the Brazilian population in the last five years, there were 195,497,797 Brazilians in 2010 and 204,450,649 Brazilians in 2015, with a percentage of 49% male and 51% female. There was an aging of the Brazilian population in this period, with an increase of 22% of the population over 65 years. Deaths from CVD represented 28% of total deaths in Brazil.

**Table 1:** Population, deaths, consultations, hospitalizations for CVD per year. Brazil, 2010-2015

**Data/Year**	**2010**	**2011**	**2012**	**2013**	**2014**	**2015**
Brazilian population	195.497.797	197.397.018	199.242.462	201.032.714	202.768.562	204.450.649
Population aged > 65 years	13.253.407	13.749.501	14.289.040	14.870.086	15.489.166	16.143.835
Total deaths	1.136.947	1.170.498	1.181.166	1.210.474	1.199.937*	1.217.673*
DCV Deaths	326.371	335.213	333.295	339.672	328.367*	334.076*
DCV SUS hospitalizations	1.153.213	1.159.210	1.137.024	1.133.235	1.140.792	1.124.156
Medical consultations DCV SUS	121.060.024	129.182.174	131.666.891	138.680.162	147.495.929	144.879.479
Surgical admissions for CVD SUS	246.038	259.888	267.323	275.838	285.109	279.010
Hospitalizations for coronary angioplasty SUS	55.980	62.221	67.113	70.744	73.939	75.410
Hospitalizations for SUS pacemaker implantation	19.937	20.857	21.959	22.448	23.426	23.300
Hospitalizations for the realization of FES SUS	5.532	6.052	6.392	6.962	6.751	7.417
Hospitalization for vascular surgeries SUS	10.238	10.719	11.586	13.403	14.931	15.907
Clinical hospitalizations for CVD SUS	874.949	870.306	844.018	831.130	819.789	807.304

Source: IBGE, SIA/DATASUS e SIH/DATASUS.

* The total number of deaths and the number of CVD deaths in the years 2014 and 2015 was estimated by calculating linear progression

There was a decrease in the number of clinical admissions due to CVD from 874,949 in 2010 to 807,304 in 2015. However, there was an increase in CVD surgical hospitalizations from 246,038 to 279,010, with a 55% increase in hospitalizations for procedures related to vascular surgeries, 35% for coronary angioplasty, and 34% for electrophysiological studies (FES).

### Direct costs

The estimated direct costs of public sector with CVD in Brazil from 2010 to 2015 are shown in Table 2. The estimated expenditures with cardiac consultations increased from approximately R$ 1.2 to R$ 1.5 billion. Expenditures on hospital admissions for CVD increased 28%, with higher expenses paid for surgical hospitalizations. The total direct expenditure on hospitalizations and consultations for CVD in 2015 was R$ 5,103,930,001.38.

**Table 2:** Values spent on consultations, hospitalizations in the SUS per CVD per year. Brazil, 2010-2015

**Data/Year**	**2010**	**2011**	**2012**	**2013**	**2014**	**2015**
Estimated cardiological medical consultations SUS	R$ 1.210.600.244,00	R$ 1.291.821.736,00	R$ 1.316.668.909,00	R$ 1.386.801.616,00	R$ 1.474.959.285,00	R$ 1.448.794.790,00
DCV SUS hospitalizations	R$ 2.094.586.170,18	R$ 2.280.690.735,84	R$ 2.381.639.909,14	R$ 2.490.327.299,45	R$ 2.616.411.987,59	R$ 2.672.683.530,36
Surgical admissions for CVD SUS	R$ 1.220.173.241,66	R$ 1.408.938.230,39	R$ 1.476.651.259,61	R$ 1.523.040.930,90	R$ 1.591.102.088,65	R$ 1.595.198.657,90
Coronary angioplasty SUS	R$ 334.006.069,71	R$ 374.975.648,22	R$ 409.312.529,41	R$ 431.199.989,60	R$ 459.208.716,25	R$ 470.525.283,05
Pacemaker / CDI SUS	R$ 239.463.794,84	R$ 255.854.307,54	R$ 271.049.370,77	R$ 283.030.018,64	R$ 305.711.764,52	R$ 314.135.570,00
Hospitalizations to carry out FES	R$ 19.133.666,93	R$ 21.114.282,38	R$ 23.329.967,02	R$ 25.291.313,54	R$ 24.932.895,30	R$ 27.324.342,93
Vascular Surgery	R$ 158.714.446,97	R$ 164.802.452,92	R$ 172.418.564,05	R$ 195.286.271,67	R$ 212.540.472,21	R$ 205.203.059,86
Clinical admissions for CVD	R$ 850.627.032,61	R$ 867.021.396,62	R$ 889.182.386,96	R$ 914.259.698,41	R$ 951.621.476,71	R$ 982.451.681,02
SUS direct expenses	R$ 4.155.813.446,79	R$ 4.439.533.868,46	R$ 4.587.491.205,10	R$ 4.791.388.613,86	R$ 5.042.992.749,30	R$ 5.103.930.001,38

Source: SIA/DATASUS and SIH/DATASUS.

The expenses related to the OPMEs were included in the amounts spent on the surgical hospitalizations in CVD in the SUS. The average values in Brazil spent on OPME were multiplied by the number of procedures with codes in the procedure table (TABNET)^20^ that use each type of material. The estimated values are shown in Table 3. The average price practiced in Brazil was taken from the table of market values published by ANVISA. The estimated expenditures in OPME increased from R$ 557,624,803.82 in 2010 to R$ 715,347,170.25 in 2015 (a 28% increase in the percentage).

**Table 3:** Amounts spent on special materials in SUS by CVD per year. Brazil, 2010-2015

**Data/Year**	**2010**	**2011**	**2012**	**2013**	**2014**	**2015**
Expenditures on coronary stents	R$ 213.729.307,50	R$ 237.557.185,46	R$ 256.234.637,63	R$ 270.097.644,33	R$ 282.296.021,21	R$ 287.912.237,92
Prosthetic expenses and vascular stents	R$ 101.859.798,52	R$ 101.813.096,10	R$ 109.033.160,55	R$ 131.874.682,40	R$ 138.307.046,37	R$ 139.058.871,31
Pacemaker costs	R$ 172.383.272,80	R$ 181.073.385,60	R$ 190.190.120,80	R$ 193.936.978,40	R$ 201.300.852,00	R$ 200.252.844,80
CDI Expenses	R$ 69.652.425,00	R$ 68.775.320,39	R$ 74.915.052,67	R$ 79.300.575,72	R$ 88.793.943,28	R$ 88.123.216,22
Total expenses with OPME	R$ 557.624.803,82	R$ 589.218.987,54	R$ 630.372.971,64	R$ 675.209.880,86	R$ 710.697.862,85	R$ 715.347.170,25

Source: Table of SIH / DATASUS procedures and material values of ANVISA.

The amounts reimbursed by the ANS to the public sector for Hospital Admissions Authorizations (AIH) and/or High Complexity Procedure Authorization (APAC) for visits to the SUS are added to the direct cost calculation. Table 4 shows the coverage rate of the ANS year by year and the expenses with reimbursements to the SUS for hospitalizations and outpatient visits of its beneficiaries. The value of the year 2015 was estimated through the calculation of linear progression, since the value is not available until the present date.

**Table 4:** ANS coverage rate and amounts spent reimbursed to SUS due ANS beneficiaries. Brazil. 2010 - 2015

**Data/year**	**2010**	**2011**	**2012**	**2013**	**2014**	**2015**
Coverage rate (%)	23,6	24	24,6	25,5	26	25,6
Amount reimbursed to SUS	R$ 78.850.898,00	R$ 74.994.805,00	R$ 88.213.668,00	R$ 84.807.361,00	R$ 96.928.835,33	R$ 103.459.587,90*

Source: ANS.

* Estimated by linear progression calculation.

The costs of the drugs used for CVD were estimated through the information collected in the transparency portal of Brazil. All direct expenditures of the federal government with pharmaceutical medicines were added.^21^ For the calculation of the percentage of total expenditure with CVD, the same percentage of 10% was used year by year.

### Indirect costs

Social Security spending on pensions and sickness-help because of CVD is available with data open on the DATAPREV portal.^12^ The data available refer to the years 2008 to 2013, without updates until the completion of the present study. The values spent in the years 2010 to 2013 in Brazil by CVD are detailed in Table 5. In order to estimate the amounts spent in the years 2014 and 2015, a statistical analysis of the previous years was made and the amount spent was estimated by linear progression.

**Table 5:** Social Security expenditures with pensions and sickness-help per CVD per year. Brazil, 2010-2015

**Data/Year**	**2010**	**2011**	**2012**	**2013**	2014*	2015*
32-Ap Disability	R$ 37.615.271,29	R$ 40.444.323,27	R$ 42.972.030,79	R$ 47.495.480,16	R$ 46.720.481,83	R$ 50.581.850,60
Ap Disability Det. Ignored	R$ 3.044.567,55	R$ 3.070.944,17	R$ 3.212.029,12	R$ 3.419.950,57	R$ 3.572.476,76	R$ 3.742.351,63
Retired due accident	R$ 760.432,36	R$ 841.083,89	R$ 847.858,46	R$ 840.913,39	R$ 1.011.029,71	R$ 1.046.865,58
Sickness-help Accident	R$ 5.243.965,12	R$ 5.298.365,35	R$ 4.958.255,43	R$ 5.447.369,98	R$ 5.034.996,01	R$ 4.860.224,76
Sickness-help	R$ 271.466.841,76	R$ 305.155.132,76	R$ 353.206.016,30	R$ 397.833.261,78	R$ 423.022.209,26	R$ 467.767.157,75
Total Social Security Expenses	R$ 318.131.078,08	R$ 354.809.849,44	R$ 405.196.190,10	R$ 455.036.975,88	R$ 479.361.193,58	R$ 527.998.450,32

Source: DATAPREV.

Table 5 shows that in 2010 R$ 318,131,078.08 were spent due to temporary or permanent removals because of CVD in Brazil. The estimated cost for 2015 is R$ 380,402,308.87. Disability pension expenses related to CVD grew exponentially. This increase cannot be justified by the Brazilian per capita GDP. The GDP per capita in 2010 of the total benefits for CVD was 1.63 and in 2015 was 1.2. The amount of benefits granted by Social Security because of CVD corresponds to 8% of total benefits granted. The number of benefits granted in CVD disability pensions has been declining in Brazil in recent years (a 10% drop), with an increase in the number of sickness-help (6% increase).

The costs of morbidity (absenteeism) are shown in Table 6. The number of admissions and medical consultations in the period from 2010 to 2015, by sex, was used to calculate the number of days lost by CVD. The number of days lost with medical appointments was halved, considering that the worker loses half of his day worked to undergo a medical consultation. The minimum cost for transportation is added to the value, considering the minimum value of public transportation for each year in the same period. The cost of morbidity in 2010 was R$ 4,264,270,533.06, while the cost of morbidity in 2015 was R$ 5,657,186,269.96

**Table 6:** Costs of CVD morbidity per year. Brazil, 2010-2015

**Data/year**	**2010**	**2011**	**2012**	**2103**	**2014**	**2015**
Hospitalizations, male	569.537	574.593	567.461	565.431	569.142	569.604
Hospitalizations, female	583.676	584.617	569.563	567.804	571.650	556.175
TMP	6,5	6,6	6,6	6,6	6,7	6,5
Days of hospitalizations, female	3.793.894	3.858.472	3.759.116	3.747.506	3.830.055	3.615.138
Days of hospitalizations, male	3.701.991	3.792.314	3.745.243	3.731.845	3.813.251	3.702.426
Consultations	121.060.024	129.182.174	131.666.891	138.680.162	147.495.929	144.879.479
Average salary (R$), female	R$ 983,37	R$ 761,00	R$ 824,00	R$ 902,00	R$ 1.000,00	R$ 895,20
Average salary (R$), male	R$ 1.390,99	R$ 1.340,00	R$ 1.430,00	R$ 1.540,00	R$ 1.664,00	R$ 1.611,36
Cost of absenteeism for hospitalizations, female	R$ 124.360.051,43	R$ 97.876.578,14	R$ 103.250.380,64	R$ 112.675.025,76	R$ 127.668.500,00	R$ 107.875.703,00
Cost of absenteeism for hospitalizations, male	R$ 171.647.725,52	R$ 169.390.016,40	R$ 178.523.230,60	R$ 191.568.022,80	R$ 211.508.344,32	R$ 198.864.705,31
Cost of absenteeism per consultation	R$ 2.395.333.996,12	R$ 2.261.764.556,11	R$ 2.473.143.100,74	R$ 2.822.141.288,56	R$ 3.274.409.612,70	R$ 3.026.242.557,35
Cost days lost due to morbidity	R$ 2.691.34.773,07	R$ 2.529.031.150,65	R$ 2.754.916.711,98	R$ 3.126.384.337,12	R$ 3.613.586.457,02	R$ 3.332.982.965,66
Minimum ticket price (R$)	R$ 2,34	R$ 2,34	R$ 2,44	R$ 2,66	R$ 2,66	R$ 3,02
Cost of Transportation (R$)	R$ 566.560.914,19	R$ 604.572.572,45	R$ 642.534.427,59	R$ 737.458.428,57	R$ 784.337.964,40	R$ 875.963.619,18
Cost Morbidity SUS	R$ 3.257.902.687,26	R$ 3.133.603.723,10	R$ 3.397.451.139,57	R$ 3.863.842.765,69	R$ 4.397.924.421,42	R$ 4.208.946.584,85
Coverage Rate	23,6	24	24,6	25,5	26	25,6
SUS + private morbidity cost	R$ 4.264.270.533,06	R$ 4.123.162.793,55	R$ 4.505.903.368,13	R$ 5.186.366.128,44	R$ 5.943.141.110,03	R$ 5.657.186.269,96

Source: SIA/DATASUS, SIH-DATASUS, PME/IBGE. TMP: mean length of stay.

Mortality costs are shown in Table 7 and were performed using the formula given in Figure 1. The years of life lost by premature death by sex are multiplied by the average salary each year, corrected for the unemployment rate in the same period. The cost of mortality reached R$ 22,275,402,229.74 reais in 2014 and was estimated at almost 22 billion reais in the year 2015.

**Table 7:** Costs of CVD mortality per year. Brazil, 2010-2015

**Data/year**	**2010**	**2011**	**2012**	**2013**	**2014**	**2015**
Women's Lost Years	520.810	527.350	511.302	512.366	517.223	516.891
Men's Lost Years	791.003	805.339	799.250	799.692	804.724	807.857
Cost of mortality (R$)	R$ 19.349.117.782,66	R$ 17.765.622.096,07	R$ 18.770.886.111,48	R$ 20.324.158.368,20	R$ 22.275.402.229,74	R$ 21.173.626.058,79

Source: SIM, PME/IBGE.

The total costs estimated with CVD in Brazil in the period from 2010 to 2015 is shown in Table 8. There was a 17% increase in CVD costs between 2010 and 2015, with an increase in the minimum salary of 55% in the same period. This means that there has been an increase in the per capita costs of the Brazilian with CVD in the last five years. The per capita expenditure in 2010 was R$ 154.41 and was estimated at R$ 172.62 in 2015. Despite this increase, the amount represented 30% of the minimum salary in 2010 and now represents 22 % in 2015.

**Table 8:** Estimated total CVD costs per year. Brazil, 2010-2015

**Data/Year**	**2010**	**2011**	**2012**	**2013**	**2014**	**2015**
Direct costs	R$ 6.169.421.794,00	R$ 6.616.780.073,69	R$ 6.920.244.266,23	R$ 7.337.716.100,29	R$ 7.775.257.583,99	R$ 7.821.609.101,66
ANS reimbursement	R$ 78.850.898,00	R$ 74.994.805,00	R$ 88.213.668,00	R$ 84.807.361,00	R$ 96.928.835,33	R$ 103.459.587,90
Cost of Morbidity	R$ 4.264.270.533,06	R$ 4.123.162.793,55	R$ 4.505.903.368,13	R$ 5.186.366.128,44	R$ 5.943.141.110,03	R$ 5.657.186.269,96
Mortality cost	R$ 19.349.117.782,66	R$ 17.765.622.096,07	R$ 18.770.886.111,48	R$ 20.324.158.368,20	R$ 22.275.402.229,74	R$ 21.173.626.058,79
Cost of pensions and helps	R$ 318.131.078,08	R$ 354.809.849,44	R$ 405.196.190,10	R$ 455.036.975,88	R$ 479.361.193,58	R$ 527.998.450,32
Cost of medication	R$ 968.489.393,60	R$ 1.286.742.089,14	R$ 2.277.654.330,14	R$ 1.418.869.356,06	R$ 2.162.470.925,74	R$ 1.819.345.140,75
Total cost DCV	R$ 31.148.281.479,40	R$ 30.222.111.706,89	R$ 32.968.097.934,08	R$ 34.806.954.289,87	R$ 38.732.561.878,41	R$ 37.103.224.609,38

The costs estimated for premature death with CVD represent 61% of total cost for CVD, direct costs were 22% and costs for morbidity were 15% of total CVD costs.

The percentage of GDP with CVD by the study estimate was 0.8% in 2010, 0.7% in the years 2011 to 2014 and 0.6% of GDP in 2015, with an average of 0.7% of GDP over the last five years.

## Discussion

It is essential today, with scarce resources, to discuss health costs based on sources of secure and real-time information.^22^ The present study has a methodology that proposes to use the data sources with greater reliability and with greater speed in obtaining the information. The sources of billing expenses in Brazil occur with a maximum delay of about 2 months after the current month, for which reason they were prioritized. It is important to point out that the present study was based on the use of the largest possible amount of information available in public databases. As the nationally based information system is still settling in Brazil, billing data on SIH and SIA may be underestimated, and this is a limitation of the study.

Another limitation of the study is information related to mortality, pensions and sickness-help, as well as the amounts of direct reimbursements from ANS to SUS. These data were not available for the years 2014 and 2015 in their fullness, in this way, it was chosen to estimate these values through linear progression. Expenses with drugs for treatment of cardiovascular disease are not available in Brazilian public information systems. The present study estimated expenditures for CVD drugs, considering that 10% of the pharmaceutical care expenses in Brazil are for CVD treatment. This strategy can be considered conservative. The criteria adopted for the selection of sources was to use as much information as possible in public databases, and to strengthen this information as a basis for the future of the country's public policies.^13,23^

The direct costs of supplementary health should be underestimated, since using the coverage rate available in the ANS assumes that the costs of supplementary medicine are at least similar to those spent in the SUS, which is known not to be a reality.

The number of CVD pensions has been decreasing in the country in the analyzed period, and the number of sickness-help has increased. This can be considered an indirect indication that the Brazilian population is living with CVD, without interruption of labor activities. This may have been due to improved health, or changes in pension legislation.

With the epidemiological transition in Brazil, associated with population aging, it is vehement that several studies estimate health costs, especially CVD, as the main cause of death due to illness in the country. As shown in the last five years, CVD costs are growing, and it is essential that health promotion measures occur to reduce premature deaths.^18,24^

## Conclusions

The direct and indirect costs of CVD in Brazil have increased in the last five years in Brazil. This increase was more significant in drug costs (88%), followed by social security costs (66%) and morbidity costs (33%). These data are indirect indications that there is an increase in the population living with CVD. The number of social security benefits has increased in the last five years in proportion to the sickness-help, since it is possible to visualize the fall of the pensions occurred by CVD. When analyzed as a percentage of GDP, CVD costs are stable, probably due to the lower number of years of life lost. The goal of the WHO was to reduce 25% of CVDNCs by 2025,^25^ and in line with the global targets for the reduction of CVD, the *Sociedade Brasileira de Cardiologia* (SBC) launched in 2013 an important publication aimed at increasing CVD prevention in Brazil.^24^ In order to achieve a reduction in the impact of CVD in Brazil, it is essential to involve everyone in society. The fight to prevent and improve the quality of life of the population is urgent, especially in a developing country such as Brazil where resources are scarce.
